# Effect of Osteocyte-Ablation on Inorganic Phosphate Metabolism: Analysis of Bone–Kidney–Gut Axis

**DOI:** 10.3389/fendo.2017.00359

**Published:** 2017-12-21

**Authors:** Osamu Fujii, Sawako Tatsumi, Mao Ogata, Tomohiro Arakaki, Haruna Sakaguchi, Kengo Nomura, Atsumi Miyagawa, Kayo Ikuta, Ai Hanazaki, Ichiro Kaneko, Hiroko Segawa, Ken-ichi Miyamoto

**Affiliations:** ^1^Department of Molecular Nutrition, Institution of Biomedical Science, Tokushima University Graduate School, Tokushima, Japan

**Keywords:** osteocyte, phosphate, fibroblast growth factor 23, Klotho, intestine, kidney, liver, bile acid

## Abstract

In response to kidney damage, osteocytes increase the production of several hormones critically involved in mineral metabolism. Recent studies suggest that osteocyte function is altered very early in the course of chronic kidney disease. In the present study, to clarify the role of osteocytes and the canalicular network in mineral homeostasis, we performed four experiments. In Experiment 1, we investigated renal and intestinal Pi handling in osteocyte-less (OCL) model mice [transgenic mice with the dentin matrix protein-1 promoter-driven diphtheria toxin (DT)-receptor that were injected with DT]. In Experiment 2, we administered granulocyte colony-stimulating factor to mice to disrupt the osteocyte canalicular network. In Experiment 3, we investigated the role of osteocytes in dietary Pi signaling. In Experiment 4, we analyzed gene expression level fluctuations in the intestine and liver by comparing mice fed a high Pi diet and OCL mice. Together, the findings of these experiments indicate that osteocyte ablation caused rapid renal Pi excretion (*P* < 0.01) before the plasma fibroblast growth factor 23 (FGF23) and parathyroid hormone (PTH) levels increased. At the same time, we observed a rapid suppression of renal Klotho (*P* < 0.01), type II sodium phosphate transporters Npt2a (*P* < 0.01) and Npt2c (*P* < 0.05), and an increase in intestinal Npt2b (*P* < 0.01) protein. In OCL mice, Pi excretion in feces was markedly reduced (*P* < 0.01). Together, these effects of osteocyte ablation are predicted to markedly increase intestinal Pi absorption (*P* < 0.01), thus suggesting that increased intestinal Pi absorption stimulates renal Pi excretion in OCL mice. In addition, the ablation of osteocytes and feeding of a high Pi diet affected FGF15/bile acid metabolism and controlled Npt2b expression. In conclusion, OCL mice exhibited increased renal Pi excretion due to enhanced intestinal Pi absorption. We discuss the role of FGF23–Klotho on renal and intestinal Pi metabolism in OCL mice.

## Introduction

Inorganic phosphate (Pi) homeostasis is maintained by complex interactions between vitamin D, parathyroid hormone (PTH), and fibroblast growth factor 23 (FGF23) (1–3). FGF23 promotes renal Pi excretion by decreasing its reabsorption in the proximal tubules while concurrently reducing plasma 1,25(OH)_2_D by both decreasing its biosynthesis and increasing its metabolism (3–6). FGF23 requires an additional cofactor, α-Klotho (Klotho), to bind with high affinity and signal efficiently through its cognate FGF receptor (7, 8). The FGF23–Klotho system directly participates in the bone–parathyroid axis as FGF23 inhibits PTH secretion (9–11). The sodium-dependent P_i_ cotransport system includes the Npt2a and Npt2c cotransporters, which locate in the apical membrane of the proximal tubular cells, and Npt2b cotransporter, which locates in the apical membrane of the intestinal epithelial cells (12–16). The FGF23–Klotho system suppresses renal Npt2a and Npt2c protein levels and decreases active vitamin D metabolism (17, 18). The reduction of plasma 1,25(OH)_2_D levels leads to decreased intestinal Npt2b protein levels (19). The FGF23–Klotho system regulates the three transporters and controls systematic Pi homeostasis (12, 13).

Elevated circulating FGF23 levels are strongly related to adverse outcomes in patients with chronic kidney disease (CKD) of all stages (20–22). Circulating FGF23 levels increase early in the course of CKD and reach levels that are several hundred times the normal range in advanced CKD and end-stage renal disease (20–22). CKD is also associated with reduced Klotho expression (23). Klotho deficiency is not only an early biomarker of CKD but also a pathogenic intermediate for CKD development and progression, and extrarenal complications (23). The causes of the increased cardiovascular risk associated with kidney disease are partly related to the CKD-mineral bone disorder (CKD-MBD) syndrome, with the FGF23–Klotho system playing an important role in the pathogenesis of CKD-MBD (23).

Osteocytes are abundant in bone and comprise 95% of all bone cells (24–26). The specialized morphology of osteocytes allows them to function effectively to direct the balance between osteoblast and osteoclast activity, and to regulate systemic mineral metabolism (24–26). Osteocytes respond to kidney damage by increasing the production of secreted factors important for bone and mineral metabolism (27). Indeed, these cells are the primary production site of several factors important for bone and mineral metabolism, including FGF23 and sclerostin (SOST), a negative regulator of the Wnt/β catenin pathway in bone (27, 28). Several recent reports suggest that altered osteocyte function is manifested by changes in osteocytic FGF23, and dentin matrix acidic phosphoprotrein-1 (DMP1) and SOST expression are observed very early in the course of CKD (27). By contrast, the decline in renal Klotho is an early event that is followed by other changes (FGF23, 1,25(OH)_2_D, PTH) as CKD progresses (29). Thus, osteocytes may be the central organ regulating Pi metabolism and dietary Pi-sensing in the kidney–bone axis (1, 24, 30, 31). The roles of osteocytes in mineral metabolism, however, have not been fully elucidated.

Recently, Tatsumi et al. established osteocyte-ablated model mice based on the diphtheria toxin receptor-mediated cell knockout (TRECK) system (32, 33) and examined the role of osteocytes in bone metabolism (32). Osteocyte-less (OCL) mice exhibit osteoporosis. Studies of osteocyte ablation model mice have provided *in vivo* evidence that osteocytes sense loading to the skeleton and orchestrate bone remodeling by controlling both osteoblasts and osteoclasts (32). The effect of osteocyte ablation on mineral metabolism remains unknown. Especially, the effects on calcium and Pi absorption, such as by the kidney and intestine, are not known. In addition, the effects of osteocyte ablation on the fluctuation of hormones related to mineral metabolism (PTH, FGF23, and vitamin D) are unclear. In the present study, we investigated mineral (calcium and Pi) metabolism in OCL mice.

## Materials and Methods

### Animal Experiments

In this study, we used the dentin matrix protein 1 promoter-diphtheria toxin receptor (DMP1-DTR) transgenic (Tg) mice established by Tatsumi et al. (32). Injecting these mice with diphtheria toxin (DT) achieves *in vivo* inducible and specific ablation of osteocytes. Ten-week-old DMP1-DTR Tg mice and wild-type (WT) mice as littermate controls (Cont) were maintained at 23°C on a 12-h light/dark cycle. The mice had unlimited access to water and a standard rodent diet. A metabolic cage was used to measure body weight, and to collect blood, urine, and feces. All animal studies were performed in accordance with the guidelines for the care and handling of laboratory animals and were approved by the animal care committee of Tokushima University.

For the dietary regulation experiments, 10-week-old male DT-injected mice (OCL and Cont mice) were placed on one of the following two isocaloric diets for 7 days: (1) control-Pi (CP) 0.6% Pi and (2) high-Pi (HP) 1.2% Pi (34, 35). For analysis of the osteocyte network-disrupted mice, we used granulocyte colony-stimulating factor (G-CSF)-injected mice. Injecting the mice with G-CSF induced hematopoietic stem/progenitor cell mobilization (36). Disruption of the osteocyte network was induced as described previously (37). Male C57 BL/6 mice were injected with recombinant human G-CSF (Filgrastim, Kyowa Kirin, 250 mg/kg body weight/day subcutaneously every 12 h in 10 divided doses unless otherwise indicated) in phosphate-buffered saline supplemented with 0.1% bovine serum albumin, and maintained for 1 day after administering the last dose of G-CSF for 24-h urine collection (37).

### Establishment of Osteocyte-Ablated Mice (OCL Mice)

**Figure 1:**
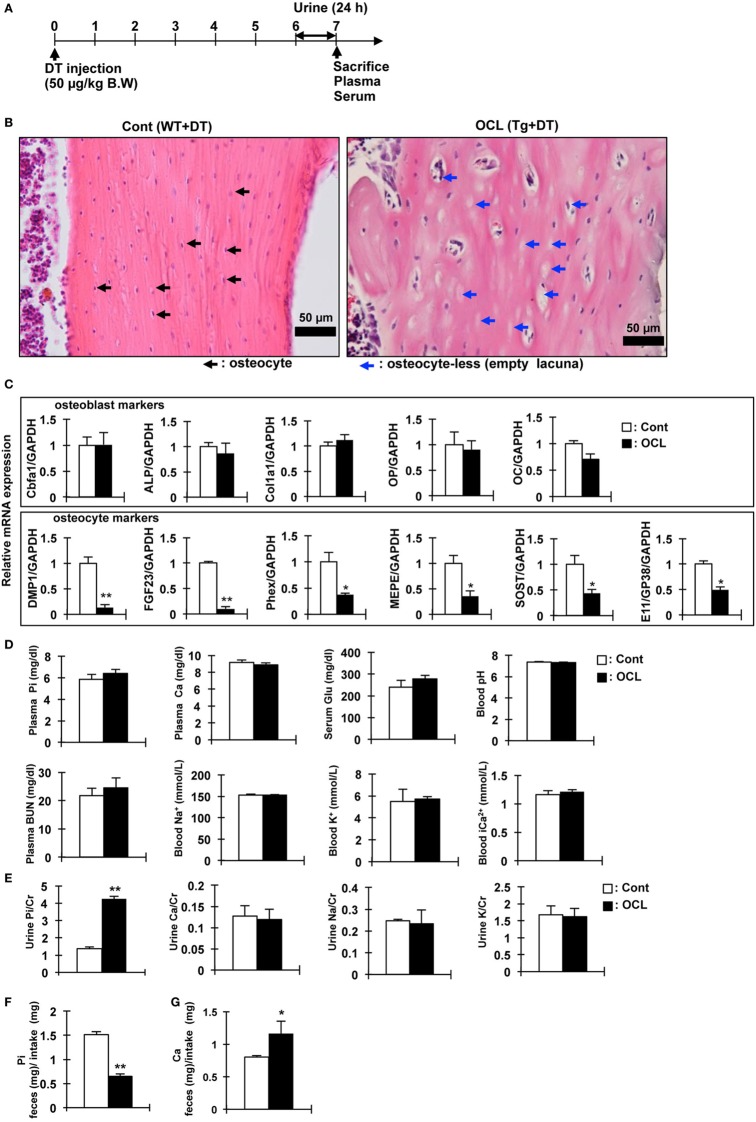
Gene expression and biochemical analysis in osteocyte-less (OCL) mice. **(A)** Experimental design. Ten-week old male wild-type (WT) and transgenic (Tg) mice were intraperitoneally injected with 50 µg/kg body weight diphtheria toxin (DT) in 0.9% NaCl were assessed at 7 days after injection. Controls (Cont): WT + DT, OCL: Tg + DT. **(B)** Cortical bone of the femurs of Cont and OCL mice stained with hematoxylin and eosin at 7 days after DT injection. Scale bars indicate 50 µm. Magnification 200×. Black arrowheads indicate osteocytes and blue arrowheads indicate ablated osteocytes (*n* = 6 mice/group). **(C)** Gene expression of osteocyte markers in femurs of OCL mice or Cont mice. Gene expression of osteocyte markers [dentin matrix protein-1 (DMP-1), fibroblast growth factor 23 (FGF23), phosphate-regulating endopeptidase homolog X-linked (Phex), matrix extracellular phosphoglycoprotein (MEPE), Sclerostin (SOST), and E11/GP38] and osteoblast markers [Cbfa1, a Alkaline Phosphatase (ALP), Collagen type I alpha (Col1a1), osteopontin (OP), and osteocalcin (OC)] were assessed by real-time polymerase chain reaction (PCR) at 7 days after DT injection. Data were normalized to glyceraldehyde 3-phosphate dehydrogenase (GAPDH) and pooled from two or three independent experiments (*n* = 6 mice/group). **(D,E)** Plasma, serum, whole blood, and urine biochemical markers in control and OCL mice at 7 days after DT injection (*n* = 6 mice/group). **(F,G)** From 6 to 7 days after DT injection, feces were collected for 24 h and food intake was measured (24 h) using a metabolic cage. **(F)** Inorganic phosphate (Pi) in feces (mg)/Pi intake (mg), **(G)** calcium in feces (mg)/Ca intake (mg) (*n* = 6 mice/group). Food consumption of the Cont mice was adjusted to that ingested by the OCL mice. Calcium and Pi levels in the feces were normalized to the amount of diet. The bar graphs are presented as arithmetic means ± SEM (*n* = 6/group). Two-tail unpaired *t*-test **P < 0.01, *P < 0.05 vs control.

DMP1-DTR Tg mice were created based on the diphtheria TRECK system (32). The mouse DMP1 gene fused to human HB-EGF (DTR) cDNA with a polyadenylation signals were constructed. The DMP1-DTR Tg mice were mated with WT mice and the offspring were genotyped by polymerase chain reaction (PCR) targeting the inserted gene using genomic DNA extracted from the tail. The following primers were used: 5′-GGGTCCCTCTTCTTCCCTAGC-3′ (forward) and 5′-GTATCCACGGACCAGCTGCTAC-3′ (reverse) (200 bp product for the transgene). Injecting DMP1-DTR Tg mice with DT resulted in targeted and inducible ablation of osteocytes. DT (50 µg/kg body weight)-treated DMP1-DTR Tg mice were used as OCL mice (Figure 1A).

### Calcium Absorption Analysis

Intestinal Ca^2+^ absorption was assessed in 8-week-old mice by measuring serum ^45^Ca^2+^ at early time-points after oral gavage. Mice were fasted 12 h before the test. Animals were hemodynamically stable under anesthesia (urethane 1.4 mg/g body weight) during the entire experiment. The test solution contained 0.1 mM CaCl_2_, 125 mM NaCl, 17 mM Tris, and 1.8 g/l fructose, and was enriched with 20 μCi ^45^CaCl_2_/ml for oral tests (Cont and OCL mice) (38). For oral tests, 15 µl/g body weight was administered orally by gavage. Blood samples were obtained at the indicated time intervals. Serum (10 µl) was analyzed by liquid scintillation counting (38). Changes in the plasma calcium concentration (Δμmol) were calculated from the ^45^Ca content of the plasma samples and the specific activity of the administered calcium (38).

### Gene Expression Studies

Total RNA was isolated from bone, liver, proximal intestine, and distal intestine with ISOGEN (Wako, Osaka, Japan). The proximal intestine refers to the duodenum and the proximal part of the jejunum. The distal intestine refers to the distal part of jejunum and ileum (39). For quantitative reverse transcription (RT)-PCR, total RNA (1 µg) was reverse-transcribed using Moloney Murine Leukemia Virus Reverse Transcriptase (Invitrogen), and samples were analyzed using the Applied Biosystems^®^StepOnePlus Real-Time PCR system (Thermo Fisher Scientific Inc., Japan). The primers and product size used are shown in Table S1 in Supplementary Material. The amount of target mRNA was normalized to that of glyceraldehyde 3-phosphate dehydrogenase (GAPDH) mRNA. RT-PCR was performed using SYBR^®^ Premix Ex Taq II (Tli RNaseH Plus) (TAKARA, OSAKA, Japan). The PCRs contained 1 µl of cDNA (equivalent to 50 ng of total RNA), 2× SYBR^®^ Premix Ex Taq II and 400 nM specific primers in a total of 25 µl. Relative expression values were evaluated with the 2^−ΔΔ^Ct method. Data were normalized to GAPDH and pooled from three independent experiments.

### Biochemical Analyses

Plasma, feces, and urinary Pi and calcium and serum blood urea nitrogen (BUN) were determined by the Phospha-C test, Calcium-E test, or BUN-B test (Wako Pure Chemical Industries, Ltd., Osaka, Japan), respectively (40). To measure the fecal Pi and calcium levels, the collected feces (24 h) were dried at 110°C for 24 h, 250°C for 3 h, and at 350°C for 3 h. FGF23 and PTH concentrations were determined using an FGF23 ELISA kit (Kainos Laboratories, Inc., Tokyo, Japan) and mouse PTH 1-84 ELISA kit (Immutopics, CA, USA), respectively (40). 1,25(OH)_2_D levels were measured using a radioreceptor assay (SRL, Inc., Tokyo, Japan). Heparinized mixed arterial–venous blood was collected and analyzed immediately for pH, blood gases, and electrolytes using an OPTI CCA TS blood gas analyzer (Sysmex Corporation, Kobe, Japan).

### Bone Analysis

Eight- to ten-week-old Tg mice and their WT littermates were perfused with 4% paraformaldehyde in 0.1 M cacodylate buffer (pH 7.4). Femurs and tibiae were immersed in the same fixative for 12 h prior to decalcification with 10% EDTA (pH 7.4) for 2 weeks. The specimens were embedded in paraffin and subjected to histochemistry with hematoxylin and eosin staining and immunohistochemical analysis using DMP1 antibody (TAKARA, Kyoto, Japan).

### Preparation of Brush Border Membrane Vesicles (BBMVs) and Transport Assay

Brush border membrane vesicles were prepared from kidney and distal intestine using the Ca^2+^ precipitation method, and used for immunoblot analysis as previously described (34, 41). Levels of leucine aminopeptidase, Na^+^-K^+^-ATPase, and cytochrome *c* oxidase were measured to assess membrane purity. Uptake of ^32^P into the BBMVs was measured by the rapid filtration technique (34, 41).

### Immunoblot Analyses

Protein samples were in denatured with 2-mercaptoethanol and subjected to 8 or 10% SDS-PAGE. The separated proteins were transferred by electrophoresis to Immobilon-P polyvinylidene difluoride (Millipore, Billerica, MA, USA) and then treated with the following diluted antibodies. Immunoblot analyses were performed using the following primary antibodies: affinity-purified anti-Npt2a (1:4,000) (34), anti-Npt2c (1:3,000) (41), and anti-Npt2b (1:2,000), as described previously (42). Anti-Klotho (for mouse total lysate; Trans Genic Inc., Fukuoka, Japan) was used following the manufacturer’s instructions. Mouse anti-actin monoclonal antibody (Chemicon, Temecula, CA, USA) was used as an internal control. Horseradish peroxidase-conjugated anti-rabbit or anti-mouse IgG was utilized as the secondary antibody (Jackson Immuno Research Laboratories, Inc., West Grove, PA, USA), and signals were detected using Immobilon Western (Millipore). Membranes were exposed to standard X-ray film and densitometric quantification was performed using ImageJ software (National Institutes of Health, Bethesda, MD, USA). All experiments were repeated at least five times.

### Histochemical Analyses of Kidney Sections

Immunohistochemical analyses of rat kidney sections were performed as described previously with minor modifications (43). Specimens were embedded in paraffin and subjected to immunohistochemistry for affinity-purified Npt2a antibodies or affinity-purified Npt2c antibodies. Sections were then treated with Envision (+) rabbit peroxidase (Dako, Carpinteria, CA, USA) for 30 min at room temperature. Immunoreactivity was detected by incubating the sections with 0.8 mM diaminobenzidine (43). Masson trichrome staining was observed using a Trichrome Stain (Masson) Kit (Sigma-Aldrich, St. Louis, MO, USA) following the manufacturer’s instructions. Von kossa stain performed in OCL and klotho-deficient mice (43). Von Kossa staining for mineral deposition in the kidney was performed by applying 5% silver nitrate to the renal sections and exposing the sections to bright light for 30 min (44). Paraffin sections were counterstained with hematoxylin for evaluation of tissue and cell morphology.

### Statistical Analysis

Data are expressed as mean ± SEM. Statistical analysis was performed using the Student’s *t*-test. We evaluated differences between the two groups by an unpaired *t*-test and differences among multiple groups were analyzed by ANOVA followed by Tukey’s post-test for multiple comparisons. All computations were performed using GraphPad Prism 5.0 Software. In all experiments, differences were considered statistically significant at *P* < 0.05.

## Results

### Pi Regulatory Genes in Bone of the OCL Mice

We investigated the physiologic function of osteocytes in OCL mice. Expression of DT receptors on osteocytes was targeted specifically using the DMP1 promoter (32). A single intraperitoneal injection of DT into DMP1-DTR Tg mice killed osteocytes harboring the DT receptor and resulted in a number of empty lacunae containing no osteocytes, as reported previously (32). The OCL mice exhibited a non-significant tendency toward decreased food intake, consistent with a previous report (45). Histochemical analysis of the cortical bone revealed a low abundance of osteocytes compared with Cont mice (32). Consistent with previous findings (9), OCL mice exhibited a decreased osteocyte number at 7 days after DT injection (Figures 1A,B). After ablated osteocytes, bone resorption was increased, bone formation and mineralization are suppressed, resulting in osteoporosis (32).

We also measured the expression of Pi regulatory genes in the osteocyte-rich fractions as described previously (32). The mRNA levels of DMP1 (*P* < 0.01), FGF23 (*P* < 0.01), matrix extracellular phosphoglycoprotein (MEPE) (*P* < 0.05), and phosphate-regulating endopeptidase homolog X-linked (Phex) (*P* < 0.05) as Pi regulatory factors were significantly decreased in OCL mice. SOST (*P* < 0.05) and E11/GP38 (*P* < 0.05) as osteocyte markers were also significantly decreased in OCL mice (Figure 1C). On the other hand, osteoblast markers were not changed in OCL mice compared with Cont mice. Compared with DT-injected WT mice, DT (50 µg/kg, DT50)-injected Tg mice showed no changes in plasma Pi, calcium, glucose, pH, BUN, Na^+^, K^+^, and Ca^2+^ (Figure 1D). In addition, urinary Ca/Cr, Na/Cr, and K/Cr concentrations were not different between DT50 mice and Cont mice (Figure 1E). By contrast, OCL mice exhibited prominent hyperphosphaturia (*P* < 0.01) (Figure 1E). Food consumption by the OCL mice was slightly lower than that of Cont mice. Food consumption by the Cont mice was adjusted to the amount in OCL mice. Calcium and Pi levels in the feces were normalized to the amount of diet consumed. The Pi content in the feces was reduced by 56.9% that in the Cont mice (*P* < 0.01) (Figure 1F). The calcium content in the feces was 45.4% higher than that in Cont mice (*P* < 0.05) (Figure 1G). These findings suggest that renal Pi excretion and intestinal Pi and calcium absorption may be abnormal in OCL mice. In particular, it is expected that the increased Pi absorption in the small intestine influences systemic Pi metabolism.

### Time-Course of the Changes in the Hormones Involved in Pi Homeostasis in OCL Mice

**Figure 2:**
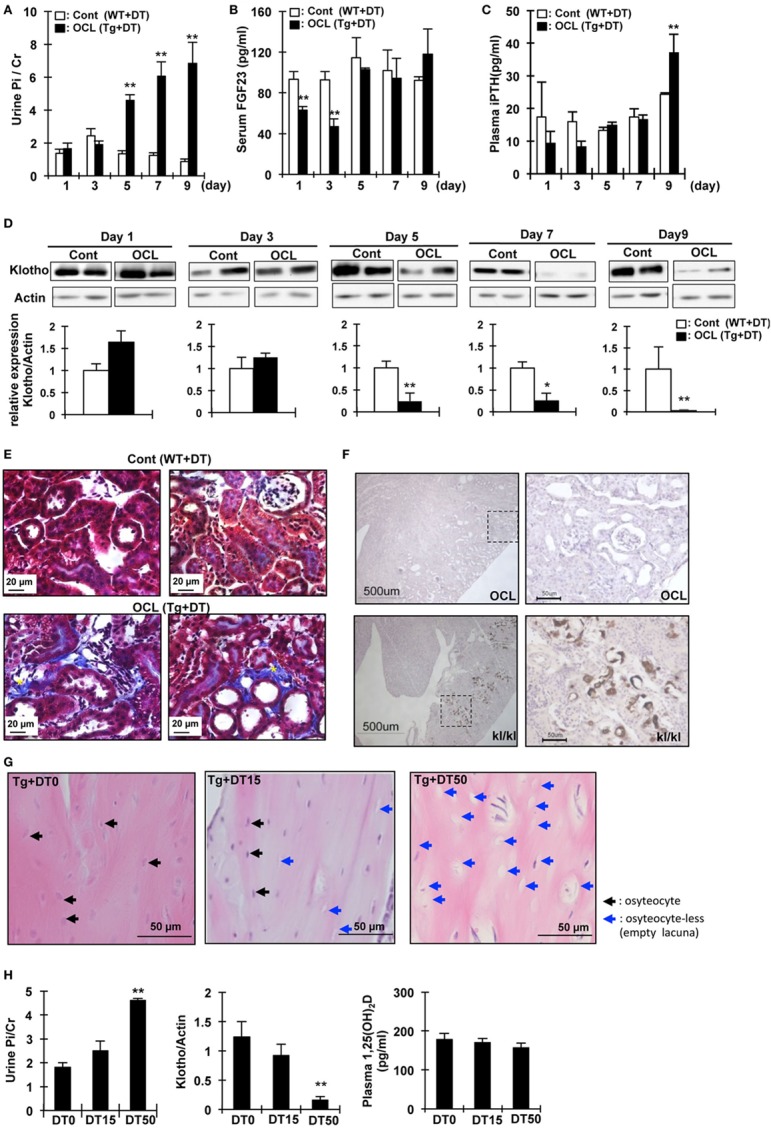
Changes in urine phosphate and phosphate-regulating factor in osteocyte-less (OCL) mice. Ten-week old male transgenic (Tg) mice and wild-type (WT) mice were intraperitoneally injected with 50 µg/kg body weight diphtheria toxin (DT) in 0.9% NaCl. Controls (Cont): WT + DT, OCL: Tg + DT. Analysis of biochemical markers at the indicated times after DT administration. **(A)** Urine Pi/Cr, **(B)** serum fibroblast growth factor 23 (FGF23), and **(C)** plasma PTH were analyzed in Cont and OCL mice after DT injection. **(D)** Renal Klotho expression in OCL mice by immunoblotting analysis (*n* = 6 mice/group). **(E)** Masson trichrome staining of sequential kidney sections from Cont and OCL mice. Scale bars indicate 20 µm. Magnification 400×. In OCL mice, severe fibrosis was observed in the peritubular interstitium. Fibrotic areas are stained blue (yellow *). **(F)** Von Kossa staining for mineral deposition in the kidney. OCL: Tg + DT (50 µg/kg body weight DT injection), kl/kl: klotho-deficient mice. Scale bars indicate 500 µm, Magnification 100× (*n* = 6/group). **(G,H)** Ten-week-old male transgenic (Tg) mice were intraperitoneally injected with 15 or 50 µg/kg body weight DT in 0.9% NaCl. DT0: vehicle (0.9%NaCl), DT15: 15 µg/kg body weight DT injection, DT50; 50 µg/kg body weight DT injection (*n* = 6/group). **(G)** Cortical bone mouse femurs stained with hematoxylin and eosin at 5 days after DT injection. Scale bars indicate 50 µm. Magnification 200×. **(H)** Urine Pi excretion in OCL. Renal Klotho expression in OCL mice by immunoblotting analysis. Plasma 1,25(OH)_2_D in OCL mice. The bar graphs are presented as arithmetic means ± SEM (*n* = 6/group). Two-tail unpaired *t*-test **P < 0.01 vs control.

To investigate the initial triggers for the increase in renal Pi excretion, we analyzed the time-course of the changes in the Pi excretion levels, plasma PTH, FGF23, and renal Klotho protein after DT treatment. Renal Pi excretion was significantly increased at 5 days after DT injection (3.45-fold, *P* < 0.01) (Figure 2A). Plasma FGF23 levels first decreased at 1 day (32.4%, *P* < 0.01) and 3 days (32.4%, *P* < 0.01) after injection of DT and then recovered (Figure 2B). By contrast, plasma PTH levels were significantly increased at 9 days (1.52-fold, *P* < 0.01) (Figure 2C). Renal Klotho levels were significantly (75.5%) decreased at 5 days after DT injection (*P* < 0.01) (Figure 2D). These data suggest that the beginning of the increase in Pi excretion is not consistent with that of the increase in plasma PTH and FGF23 levels. We analyzed renal fibrosis (Figure 2E). High dietary Pi loading increases renal fibrosis (46). In OCL mice, kidney samples were obtained at 7 days after DT injection. The kidney samples obtained from OCL mice were stained with Masson’s trichrome to detect total collagen deposits (Figure 2E), which revealed interstitial fibrosis and increased renal Pi excretion independent of plasma PTH and FGF23 levels. The kidney calcification observed in Klotho-deficient mice was not detected in OCL mice (Figure 2F).

We then investigated if DT injection induced dose-dependent changes in the osteocyte number and renal Pi excretion (32). At 5 days after DT (0, 15, or 50 µg/kg BW) injection, we measured Pi excretion and renal Klotho and 1,25(OH)_2_D levels (Figure 2G). At 5 days after DT injection, the number of osteocytes in the cortical bone decreased to 33.5 ± 1.7% (DT15) or 75.3 ± 4.2% (DT50) that in the DT0 mice (*P* < 0.01) (Figure 2G). The plasma 1,25(OH)_2_D levels did not change after DT injection (Figure 2H). Urinary Pi excretion was significantly increased at 5 days after DT50 injection (*P* < 0.01) (Figure 2C). By contrast, DT50 injection markedly suppressed renal Klotho expression (*P* < 0.01) (Figure 2D). These data suggest that the increased renal Pi excretion was dependent on the number of osteocytes ablated.

### Renal and Intestinal Pi Transport Activity in the OCL Mice

**Figure 3:**
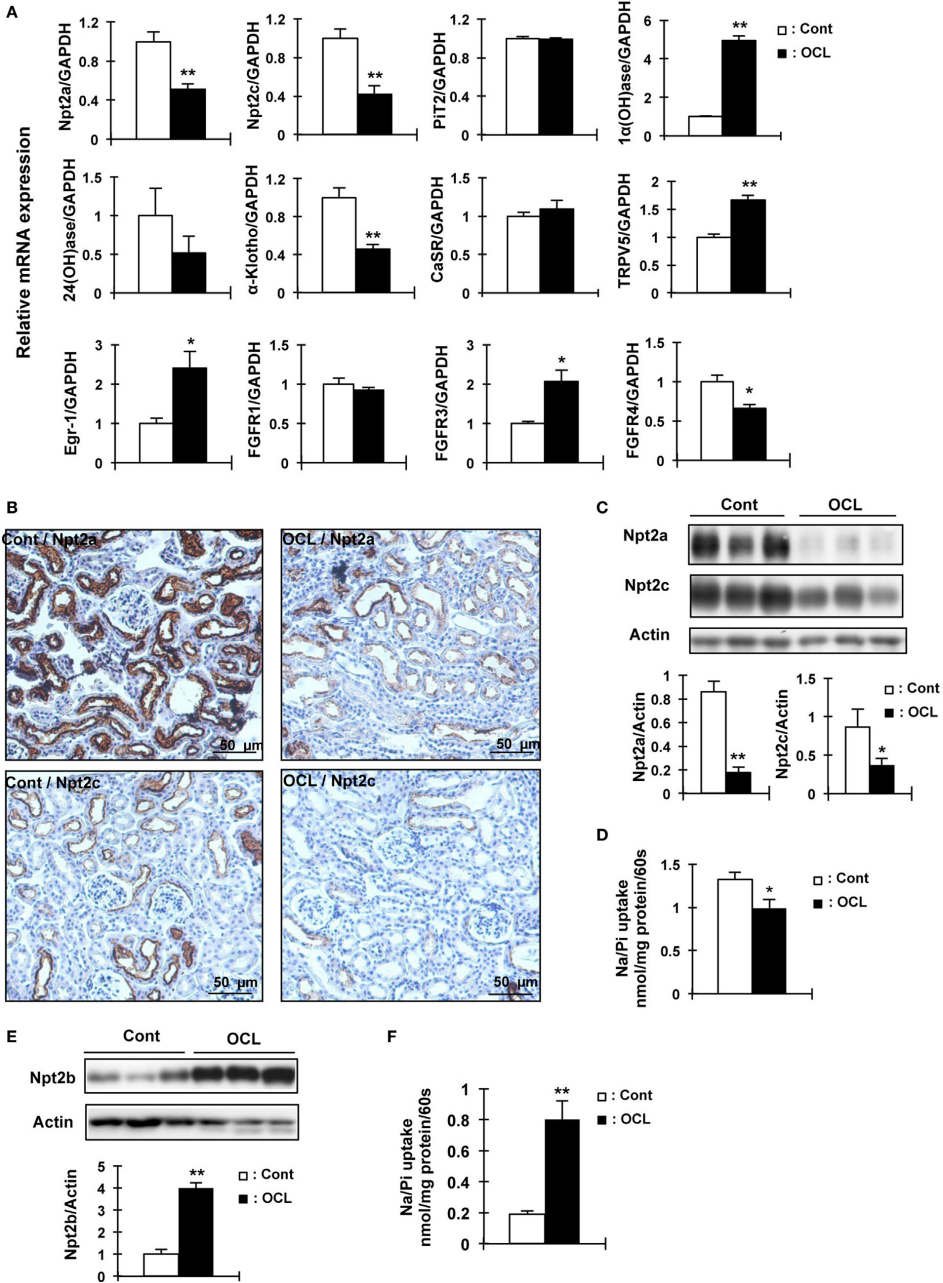
Expression of renal Npt2a, Npt2c, and intestinal Npt2b in osteocyte-less (OCL) mice. Ten-week-old male wild-type (WT) and transgenic (Tg) mice were intraperitoneally injected with 50 µg/kg body weight DT in 0.9% NaCl and assessed at 5 days after injection. Controls (Cont): WT + DT, OCL: Tg + DT. **(A)** Quantitative polymerase chain reaction (PCR) of Npt2a, Npt2c, PiT2, 1α(OH)ase, 24(OH)ase, Klotho, CasR, TRPV5, early growth response 1 (Egr-1), FGF receptor 1 (FGFR1), FGFR3, and FGF receptors 4 (FGFR4) mRNA in mouse kidney at 5 days after DT injection. Data were normalized to glyceraldehyde 3-phosphate dehydrogenase (GAPDH) and pooled from two or three independent experiments (*n* = 7 mice/group). **(B)** Immunohistochemical analysis of renal Npt2a and Npt2c proteins. Scale bars indicate 50 µm. Magnification 200×. **(C)** Immunoblotting analysis of Npt2a and Npt2c proteins in renal brush-border membrane vesicles (BBMVs). All membranes were reprobed for actin. Actin was used as an internal control. The bar graphs are presented as arithmetic means ± SEM (*n* = 5/group). **(D)** Renal Na/Pi transport activity in mice. Na/Pi transport activity was determined by ^32^P uptake in kidney BBMVs (*n* = 7/group). **(E)** Immunoblotting analysis of Npt2b proteins in intestinal (jejunum and ileum) brush-border membrane vesicles. All membranes were reprobed for actin. Actin was used as an internal control (*n* = 7 each/group). **(F)** Intestinal (distal jejunum and ileum) Na/Pi transport activity in mice. Na/Pi transport activity was determined by ^32^P uptake in intestinal BBMVs (*n* = 7/group). The bar graphs are presented as arithmetic means ± SEM, Two-tail unpaired *t*-test **P < 0.01, *P < 0.05 vs control.

To examine whether the expression of renal Pi transporters was affected in OCL mice, Npt2a and Npt2c mRNA and protein levels were investigated (Figure 3). Npt2a and Npt2c mRNA levels were significantly decreased in OCL mice (*P* < 0.01) (Figure 3A). PiT2 mRNA levels were not changed in OCL mice compared with Cont mice (Figure 3A). 1α(OH)ase mRNA levels were markedly increased in OCL mice (4.9-fold, *P* < 0.01), whereas 24(OH)ase mRNA expression levels were not changed (54.4% decreases, *P* < 0.01) (Figure 3A). Klotho mRNA expression levels were significantly (44.4%) decreased in OCL mice (*P* < 0.01) (Figure 3A).

The mRNA levels of the transcription factor early growth response 1 (Egr-1) were significantly increased in OCL mice (2.4-fold, *P* < 0.01), whereas FGF receptor 1 (FGFR1) mRNA levels were not changed. The mRNA expression levels of FGF receptors 3 (FGFR3) were significantly increased (2.1-fold, *P* < 0.01) and FGF receptors 4 (FGFR4) were significantly decreased (34%, *P* < 0.01) (Figure 3A). Npt2a and Npt2c protein levels were markedly decreased in OCL mice (*P* < 0.01, *P* < 0.05) (Figure 3C). In addition, sodium-dependent Pi cotransport activity in the BBMVs was also significantly reduced in OCL mice compared with Cont mice (*P* < 0.01) (Figure 3D). Immunohistochemical analysis revealed reduced Npt2a and Npt2c immunoreactivity in the proximal tubular cells (Figure 3B). Intestinal Npt2b protein levels were significantly increased in OCL mice compared with Cont mice (fourfold, *P* < 0.01) (Figure 3E). Intestinal Pi transport activity in OCL mice was 4.2-fold higher than that in the Cont mice (*P* < 0.01) (Figure 3F).

### Intestinal Calcium Absorption

We then investigated calcium absorption in the small intestine. In contrast to intestinal Pi absorption, the calcium content in the feces in OCL mice was 45.4% higher than that in Cont mice (*P* < 0.05) (Figure 1G).

**Figure 4:**
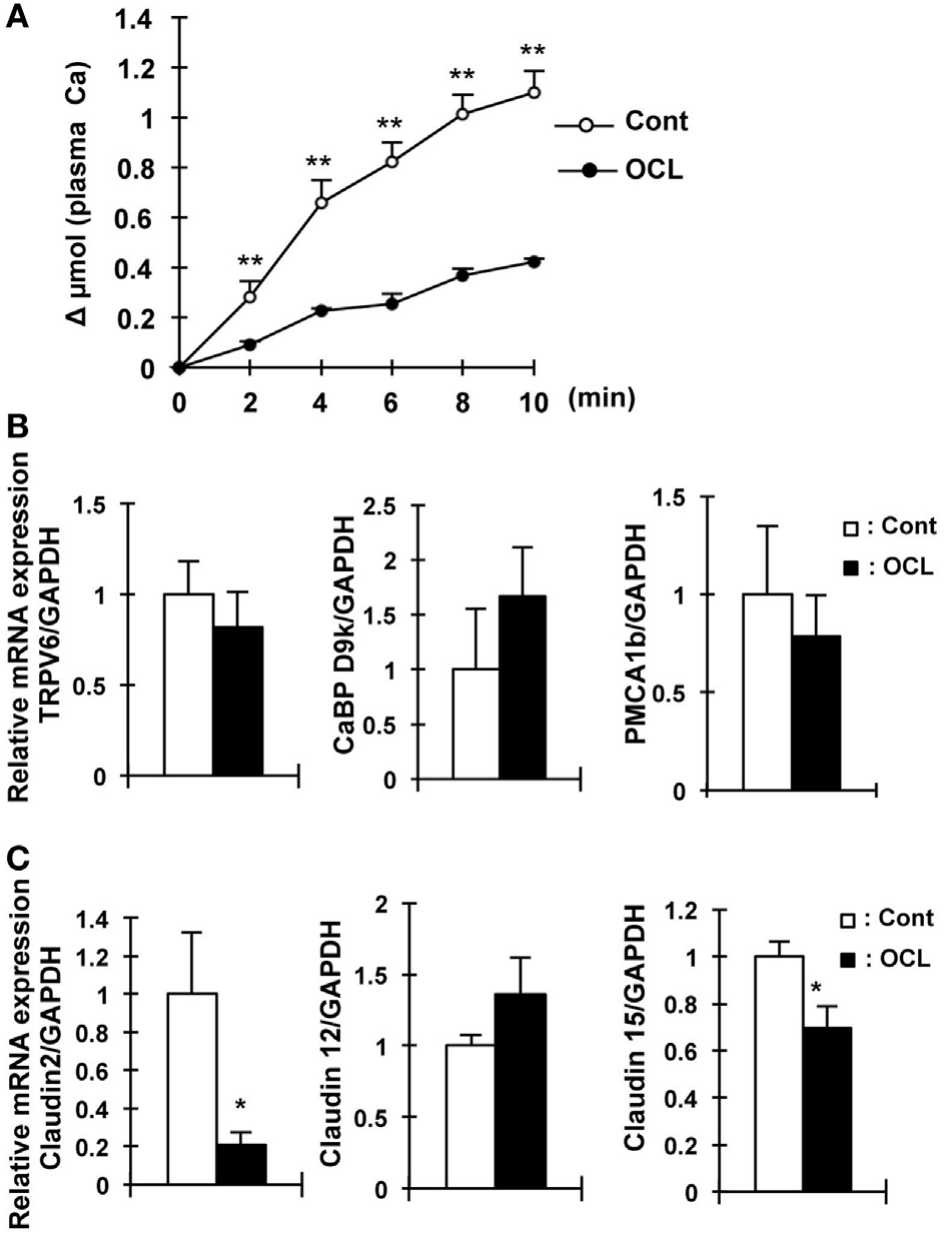
Calcium absorption in osteocyte-less (OCL) mice. Ten-week-old male wild-type (WT) and transgenic (Tg) mice were intraperitoneally injected with 50 µg/kg body weight DT in 0.9% NaCl at 5 days after DT injection. Control (Cont): WT + DT, OCL: Tg + DT. **(A)** Intestinal calcium absorption, assessed by whole blood levels of ^45^Ca (Δμmol) after oral gavage (*n* = 5/group). **(B)** Gene expression of calcium transport and its regulating factor in intestinal of OCL mice or Cont mice. Gene expression of transient receptor potential vanilloid channel 6 (TRPV6) as apical calcium transport, calbindin-D_9k_ (CaBP-9k) as cytoplasmic calcium-binding proteins, and plasma membrane calcium ATPase 1b (PMCA1b) as basolateral calcium transporter were assessed by real-time polymerase chain reaction (PCR) at 5 days after DT injection. Data were normalized to glyceraldehyde 3-phosphate dehydrogenase (GAPDH) and pooled from two or three independent experiments (*n* = 7 mice/group). **(C)** Gene expression of intestinal claudin 2, 12, and 15 in OCL mice or Cont mice. Gene expression of intestinal (proximal part of intestine) claudin 2, 12, and 15 were assessed by real-time PCR at 5 days after DT injection. Data were normalized to GAPDH and pooled from two or three independent experiments (*n* = 7 mice/group). The bar graphs are presented as arithmetic means ± SEM, two-tail unpaired *t*-test **P < 0.01, *P < 0.05 vs control.

Oral calcium absorption analysis using ^45^Ca^2+^ solution indicated that calcium absorption (plasma ^45^Ca^2+^) levels were significantly (62–69%) decreased in OCL mice (*P* < 0.01) (Figure 4A). Intestinal transient receptor potential vanilloid 6 (TRPV6), calbindin-D9k (CaBP D9k), and plasma membrane calcium ATPase 1b (PMCA1) mRNA levels, however, were not altered in OCL mice (Figure 4B). On the other hand, mRNA expression levels of claudin 2, which regulates intestinal Ca^2+^ absorption *via* paracellular pathways, were dramatically (79.1%) decreased in OCL mice (*P* < 0.01) (Figure 4C). Although claudin 12 mRNA expression levels were not changed in OCL mice, claudin 15 mRNA expression levels were decreased in OCL mice (30.4% decreases, *P* < 0.05). Thus, intestinal calcium absorption was significantly decreased in OCL mice. On the other hand, renal excretion of calcium was not affected in OCL mice. In the kidney, calcium-sensing receptor mRNA levels were not changed and TRPV5 mRNA expression was significantly (1.7-fold) increased in OCL mice (*P* < 0.01) (Figure 3A).

### Analysis of Osteocyte Canalicular Network-Disrupted Mice

**Figure 5:**
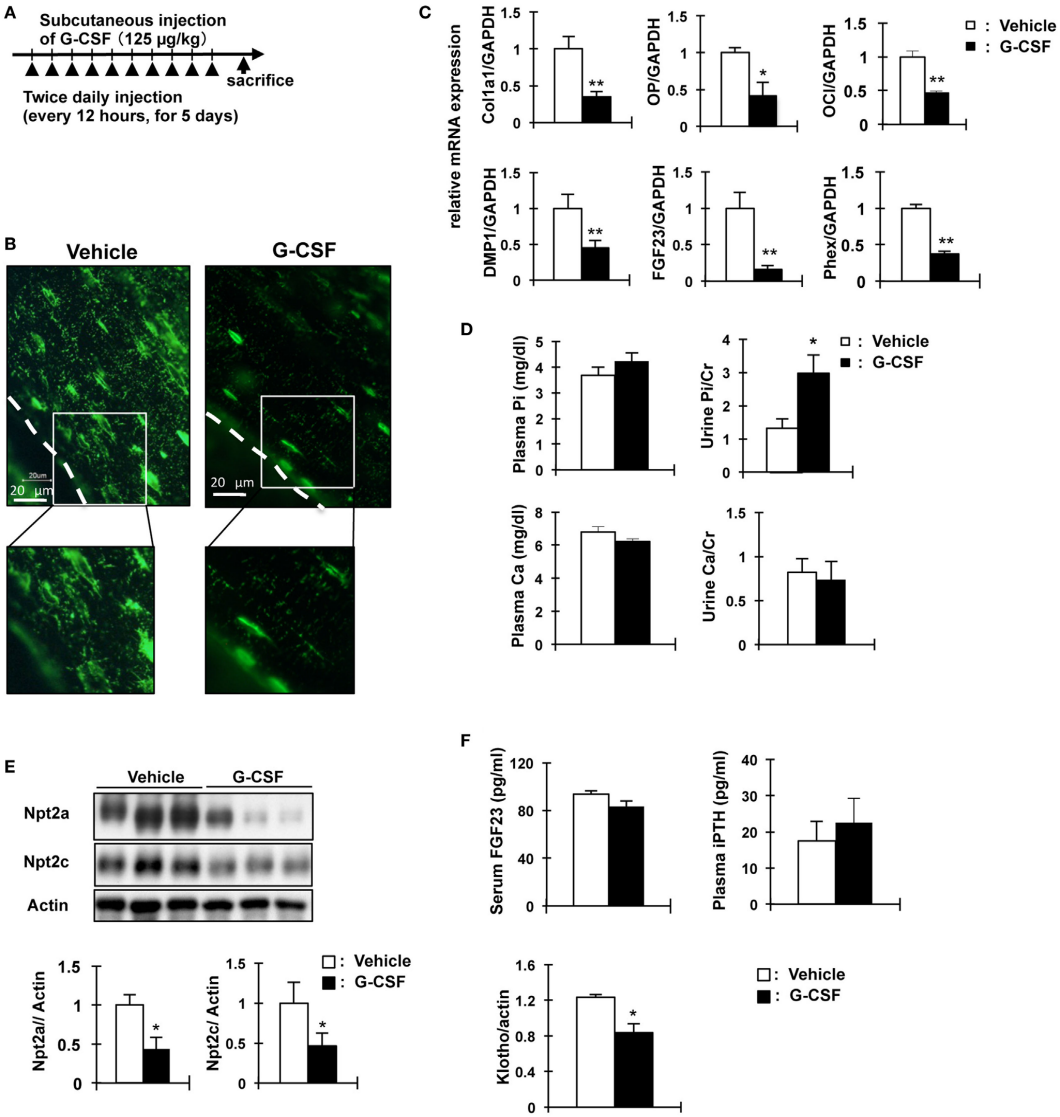
Analysis of granulocyte colony stimulating factor (G-CSF)- injected mice in phosphate metabolism. **(A)** Experimental design for osteocytic canaliculi network disruption by G-CSF (filgrastim 125 mg/kg/dose, s.c.). **(B)** Immunofluorescence staining of dentin matrix acidic phosphoprotrein-1 (DMP1) in osteocytes and lacuna-canaliculi network of the femur of vehicle and G-CSF injected mice. Green: DMP1. The scale bars indicate 20 µm. Magnification 400×. **(C)** Real-time polymerase chain reaction (PCR) analysis of markers of osteoblast and osteocytes. RNA was extracted from the femurs of 8-week-old WT mice injected with phosphate-buffered saline (white bars) or G-CSF (black bars) for 6days. Gene expression levels were normalized to glyceraldehyde 3-phosphate dehydrogenase (GAPDH) mRNA expression levels. Collagen type I alpha (Col1a1), osteopontin (OP) osteocalcin (OC), FGF23, DMP1, and Phex. Data are presented as mean ± SEM. **P < 0.01 **P < 0.05 (*n* = 6/group). **(D)** Biochemical analysis of plasma Inorganic phosphate (Pi), plasma calcium, urinary Pi/Cr, and urinary Ca/Cr levels in G-CSF or vehicle-injected mice. Urine was collected for 24 h from days 5 to 6. **(E)** Immunoblotting analysis of Npt2a and Npt2c proteins in renal brush-border membrane vesicles of G-CSF-treated mice and vehicle-injected mice. All membranes were reprobed for actin. Actin was used as an internal control. The bar graphs are presented as arithmetic means ± SEM (*n* = 6 each group). **(F)** Serum FGF23 and plasma iPTH and renal Klotho levels in G-CSF treated mice and vehicle-injected mice. **P < 0.01; *P < 0.05 (*n* = 6 mice/group). Renal Klotho protein levels were observed by immunoblotting analysis in renal total lysates. All membranes were reprobed for actin. Actin was used as an internal control. The bar graphs are presented as arithmetic means ± SEM (*n* = 6/group). Two-tail unpaired *t*-test **P < 0.01, *P < 0.05 vs control (vehicle injection).

We investigated other mouse models of osteocyte network disruption. Eight-week-old C57BL/6 mice were administered recombinant human G-CSF (250 µg/kg body weight/day) and analyzed 1 day after the last dose of G-CSF (Figure 5A). In the G-CSF injected mice, hematopoietic stem cells were removed from the medullary cavity (data not shown). RNA was extracted from the tibias and femurs of vehicle- and G-CSF-treated mice after flushing out the bone marrow. Gene expression was assessed by quantitative RT-PCR and the results were normalized to GAPDH mRNA. In G-CSF injected mice, Col1a1 (64.6%, *P* < 0.01), osteocalcin (53.8%, *P* < 0.01), and osteopontin mRNA (58.8%, *P* < 0.05) as osteoblastic markers were decreased; and FGF23 (83.8%), DMP1 (54.8%), and Phex (62.4%) as osteocytic markers were also decreased (*P* < 0.01), suggesting that differentiation of osteoblasts into osteocytes was suppressed by G-CSF injection (Figure 5C). Immunohistochemical analysis using DMP1 antibody showed that the length and number of canaliculi were markedly decreased in the femoral bone of G-CSF treated mice (Figure 5B). Remarkably, canaliculi did not reach the surface of the bone (Figure 5C). Therefore, these results indicate that the osteocytic canalicular network was disrupted. The serum Pi concentration did not significantly differ between the G-CSF-injected mice and vehicle-injected mice (Figure 5D) and was similar to that in the OCL mice (Figure 1D). Urinary Pi levels, however, were significantly higher (2.2-fold, *P* < 0.05) in the G-CSF-injected mice than in the vehicle-injected mice (Figure 5D), same as in OCL mice (Figure 1E). In the renal BBM of G-CSF mice, Npt2a and Npt2c levels were significantly decreased (*P* < 0.05) (Figure 5E), similar to the renal Npt2a and Npt2c protein levels in OCL mice (Figure 3C). In G-CSF-injected mice, plasma iPTH and FGF23 levels did not differ significantly from those in the vehicle-injected mice (Figure 5F). On the other hand, renal Klotho protein levels were decreased in G-CSF-injected mice (*P* < 0.05) (Figure 5F). Thus, mice with G-CSF-induced osteocyte network disruption showed hyperphosphaturia independent of the plasma PTH and FGF23 levels.

### Feeding on a High Pi Diet Does Not Stimulate Renal Pi Excretion in OCL and G-CSF Mice

**Figure 6:**
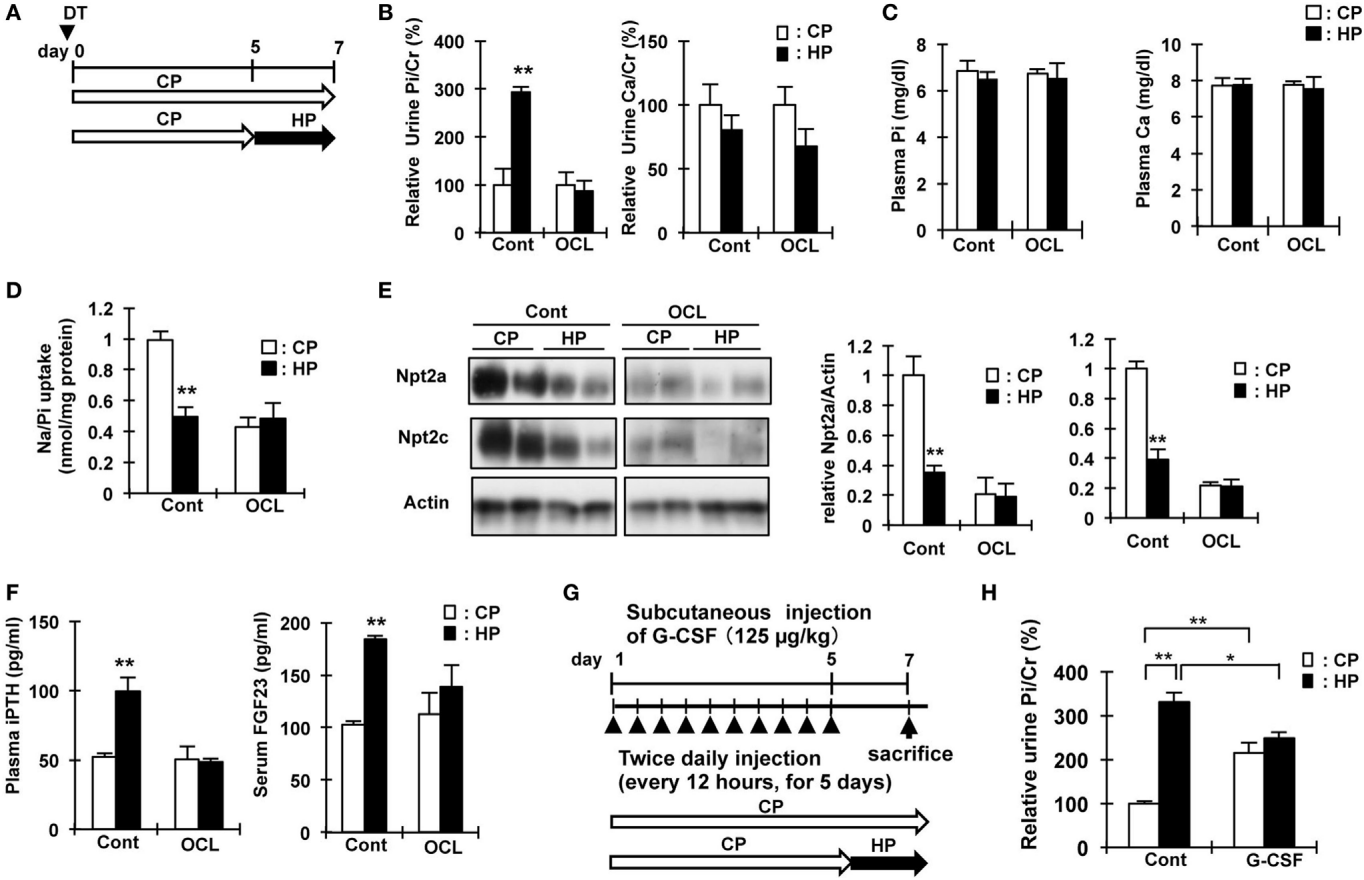
High phosphate diet response in osteocyte-less (OCL) and granulocyte colony stimulating factor (G-CSF) mice. **(A)** Experimental design of high-Pi (HP) dietary loading in OCL mice. Ten-week-old male transgenic (Tg) mice and wild-type (WT) mice were intraperitoneally injected with 50 µg/kg body weight DT in 0.9% NaCl. Control (Cont): WT + DT, OCL: Tg + DT. Control and OCL mice were fed the control inorganic phosphate (Pi) diet until day 5 and from days 5 to 7 were fed either the high Pi diet (HP) or control diet (CP). **(B)** Relative urine Pi/Cr (%) and Ca/Cr (24 h urine collection) at 6–7 days after diphtheria toxin (DT) injection. **(C)** Plasma Pi and calcium at 7 d after DT injection. **(D)** Renal Na/Pi transport activity in mice. Na/Pi transport activity was determined by ^32^P uptake in kidney brush border membrane vesicles (BBMVs) (*n* = 6/group). **(E)** Immunoblotting analysis of Npt2a and Npt2c proteins in renal BBMVs. All membranes were reprobed for actin. Actin was used as an internal control. **(F)** Plasma intact PTH and serum FGF23 at 7 days after DT injection. The bar graphs are presented as arithmetic means ± SEM (*n* = 6/group). Two-tail unpaired *t*-test **P < 0.01, *P < 0.05. (CP VS HP). **(G)** Experimental design of HP dietary loading in G-CSF mice. Seven-week-old male mice were injected with PBS or G-CSF (filgrastim 125 mg/kg/dose, s.c.). Control (Cont): WT + PBS, G-CSF: WT + G-CSF. Control and OCL mice were fed the control Pi diet until day 5 and fed either the high Pi diet (HP) or control diet (CP) from days 5 to 7. **(H)** Relative urine Pi/Cr (%) (24 h urine collection) at 6–7 days. The bar graphs are presented as arithmetic means ± SEM (*n* = 6/group). ANOVA followed by Tukey’s post-test for multiple comparisons. **P < 0.01, *P < 0.05 (CP vs HP, Cont vs G-CSF).

The increased renal Pi excretion in OCL and G-CSF mice may be due to the increase in Pi released from the bone and intestinal Pi absorption. Therefore, we investigated whether feeding mice a high Pi diet increases urinary Pi excretion. Figures 6A,G show the schedule for dietary phosphate loading in OCL and G-CSF mice. After feeding on a high Pi diet, Pi excretion was significantly increased in the Cont mice (*P* < 0.01), but not changed in the OCL and G-CSF mice (Figures 6B,H). After feeding on a high Pi diet, plasma Pi and calcium levels were not changed in the OCL mice (Figure 6C).

In Cont mice, feeding on a high Pi diet significantly reduced renal Npt2a and Npt2c protein levels (*P* < 0.01) and Na/Pi uptake in BBMs (*P* < 0.01), whereas no changes were observed in OCL mice (Figures 6D,E). In the mice fed a high Pi diet, the levels of plasma intact PTH were not changed in OCL mice, but significantly increased in Cont mice (1.9-fold, *P* < 0.01) (Figure 6F). The increase in plasma PTH levels and serum FGF23 levels induced by a high Pi diet in Cont mice was not observed in OCL mice. Therefore, for dietary Pi adaptation, signaling between the intestine and parathyroid gland, and between the intestine and kidney may be disturbed in OCL mice.

### Analysis of Gene Expression Profiles in OCL Mice

We performed DNA microarray analysis of the intestine, liver, and kidney in OCL mice (data not shown). In these analyses, we focused on bile acid metabolism, a commonly changing metabolic system, in OCL mice and G-CSF-administered mice, because we did not detect prominent differences in the expression of genes related to glucose, amino acid, other nutrient metabolic pathways. Bile acid metabolism was markedly disturbed in the intestine and liver in OCL mice compared with Cont mice (Figure S1 in Supplementary Material). In a previous study, Sato et al. reported that osteocytes regulate fat metabolism, that OCL mice lack visible white adipose tissue, and plasma leptin levels decrease in association with fat loss (45). Bile acids serve as ligands for the nuclear receptor farnesoid X receptor (FXR) (46, 47). Bile acids act on FXR in ileal enterocytes to induce the expression of FGF15 (47, 48). FGF15 also stimulates gallbladder filling (47, 48). FGF15 inhibits bile acid synthesis by repressing the transcription of Cyp7al, which encodes the first and rate-limiting enzyme in the classic bile acid synthesis pathway (43). FGF15 acts through a cell surface receptor complex composed of the FGF receptor 4 and β-Klotho (49, 50). In liver, transcription of the small heterodimer partner (SHP) gene is induced by bile acids *via* FXR, and SHP, in turn, binds to the CYP7A1 promoter to repress gene transcription (47). SHP is required for FGF15 to efficiently repress bile acid synthesis (48). In OCL mice compared with Cont mice, transcripts of FXR (liver: *P* < 0.05, distal intestine: *P* < 0.01), Cyp7A1 (*P* < 0.05), FGF receptor 4 (FGFR4) (*P* < 0.05), β-Klotho (*P* < 0.01), SHP (distal intestine; *P* < 0.01), and FGF15 (*P* < 0.01), the concentrations of serum bile acid (*P* < 0.05), and feces bile acids (*P* < 0.05) intestinal bile acids (*P* < 0005) were significantly increased in the liver and/or distal intestine compared with Cont mice (Figures S1B–D in Supplementary Material). Intestinal vitamin D receptor, calcium-sensing receptor, and parathyroid hormone 1 receptor mRNA levels were not changed in OCL mice (data not shown).

### Analysis of Gene Expression Profiles in High Pi Diet-Fed Mice

Next we investigated whether OCL mice are in a high Pi state. Lipid accumulation in white adipocytes was decreased in OCL and high Pi diet-fed mice (Figures S1A and S2A in Supplementary Material). We observed alterations in the genes involved in bile acid metabolism (*P* < 0.01) (Figure S1 in Supplementary Material). Quantitative PCR analysis revealed that, compared with Control Pi-fed mice (CP mice), high Pi-fed mice (HP mice) had significantly lower transcript levels of Cyp7A1 (*P* < 0.01), Cyp8b1 (*P* < 0.01), FGFR4 (*P* < 0.05), and β-Klotho (*P* < 0.05) in the liver. FXR (*P* < 0.01), OST-α (*P* < 0.05), and FGF15 (*P* < 0.01) levels in the distal intestine were also significantly decreased in HP mice (Figures S2B,C in Supplementary Material). On the other hand, the concentrations of serum bile acid and feces bile acids were not changed in the liver or distal intestine in HP mice compared with CP mice (Figure S2D in Supplementary Material). The serum triglyceride and cholesterol concentrations were lower in HP mice than in CP mice (Figure S2D in Supplementary Material).

## Discussion

**Figure 7:**
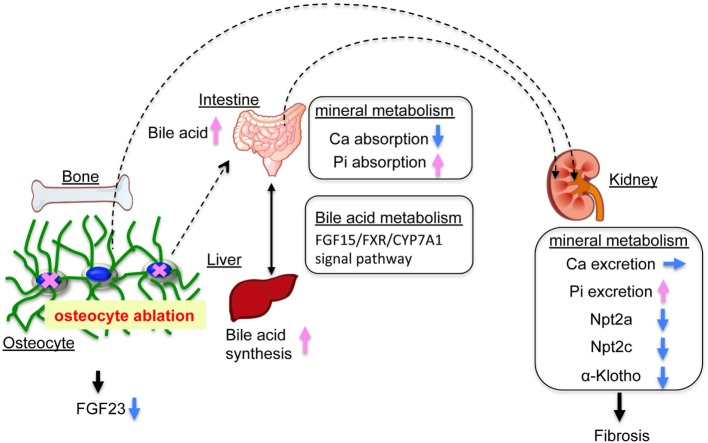
Mineral balance in osteocyte-less (OCL) mice. Osteocyte ablation affects mineral metabolism in the kidney and intestinal tract. The OCL mice showed increased renal inorganic phosphate (Pi) excretion (suppression of Npt2a and Npt2c) by enhanced intestinal Pi absorption (elevation of Npt2b). The OCL mice exhibited markedly suppressed renal Klotho expression, possibly reflecting a disrupted FGF23–Klotho system. OCL causes abnormalities in the bile acid (FGF15/FXR/CYP7A1) signaling pathway in the intestine–liver axis and stimulates intestinal Pi absorption. Thus, osteocytes may be an important regulator of mineral balance in addition to bile acid metabolism.

In the present study, we investigated the role of osteocytes in Pi metabolism using OCL mice. In this experiment, osteocyte ablation revealed that renal Pi excretion was enhanced before the plasma PTH and FGF23 levels increased. In particular, renal Klotho protein levels declined already in the early stage. In OCL mice, Pi excretion in the feces was markedly reduced and intestinal Pi absorption (Npt2b levels and Pi uptake in BBMV) was significantly enhanced. The present study indicates that the increased renal Pi excretion is due to increased intestinal Pi absorption in OCL mice (Figure 7). Similar findings regarding Pi metabolism were obtained in canalicular network-disrupted mice. Furthermore, the response to dietary Pi loading was suppressed in OCL mice.

With regard to the mechanisms underlying the suppression of renal Pi transport, we observed that renal Npt2a and Npt2c protein levels were significantly decreased in the OCL mice. The transcription factor Egr-1 is downstream of ERK1/2 and is a biomarker of ERK1/2 signaling activation by FGF23 (7). Renal Egr-1 mRNA levels were significantly increased, while Klotho protein levels were markedly decreased (7). Renal 1alpha-hydroxylase (1α(OH)ase) mRNA was upregulated in the OCL mice, suggesting that klotho downregulation is involved in the stimulation of 1,25(OH)_2_D synthesis.

Recent studies demonstrated that FGFR1c/Klotho is the primary receptor complex responsible for mediating Pi metabolism (51), whereas there is a redundant requirement for FGFR3/Klotho and FGFR4/Klotho in the control of 1,25 (OH)_2_D levels (52, 53). Thus, the two pathways are expected to be involved in vitamin D and Pi transport by the FGF23–Klotho system (6, 54). Portale et al. reported that suppression of renal 1,25(OH)_2_D synthesis was intact in egr-1^−/−^ mice treated with FGF23 (55). They found that downstream of FGFR binding and ERK1/2 signaling, the pathways diverge such that egr-1 is required for FGF23-dependent inhibition of Pi transport but not for inhibition of 1,25(OH)_2_D synthesis (55, 56). The mechanisms underlying the promotion of renal Pi excretion in OCL mice remain unknown, but the increase in egr-1 mRNA suggests the involvement of FGF23-dependent Pi inhibition. We are currently studying the molecular mechanism of the enhancement of Pi excretion in OCL mice.

Osteocyte-less (OCL) mice are thought to have abnormally high Pi absorption from the small intestine. If this hypothesis is correct, OCL mice would be in a state of high Pi and, thus, it is expected that the response of renal Pi excretion to a high Pi diet would be attenuated (43). Osteocytes extend dendrites to each other in the osteocytic canaliculi, and osteoblasts and osteoclasts connect with each other on the bone surface by gap junctions, referred to as the osteocytic canalicular network (24, 57–61). The network is complicated and extends through the entire bone, possibly forming gap junctional intercellular communication pathways (24, 57–61). Osteocyte canalicular networks are thought to be important for bone formation as the structure seems to be ideal for sensing mechanical stress and mechanotransduction (24, 57–61). A recent study demonstrated that osteocytic canalicular network-disrupted mice are created by injection of G-CSF (37). Disruption of the osteocyte canalicular network by G-CSF increased Pi excretion and decreased renal klotho levels independent of PTH and FGF23. In addition, in G-CSF-treated mice or OCL mice, renal Pi excretion did not increase in response to a high Pi diet. These observations suggest that OCL mice already had a high Pi state, and excessive Pi from intestinal absorption may promote renal Pi excretion.

In the present study, OCL mice exhibited abnormal intestinal Pi and calcium absorption. Previous studies demonstrated that the kidney rapidly increases Pi excretion when intestinal Pi absorption is markedly elevated (62, 63). In these conditions, the increase in the plasma PTH or FGF23 levels leads to increased renal Pi excretion in response to an increase in intestinal Pi absorption (62, 63). In OCL mice, however, we observed no increase in PTH or FGF 23. Therefore, the phosphaturic factors acting on the kidney are unknown. In OCL mice, 1,25(OH)_2_D may be a factor that enhances intestinal Pi absorption. With respect to the FGF23–Klotho system, plasma 1,25(OH)_2_D levels are an important factor for intestinal mineral absorption (3). In the OCL mice, plasma 1,25(OH)_2_D levels were not changed while renal 1α(OH)ase mRNA was significantly increased. We investigated the mechanism of the reduction of calcium absorption and the elevation of Pi absorption. To clarify intestinal Pi absorption, we measured intestinal Pi transport activity in BBMV and found the elevation of Npt2b protein and Pi uptake in the OCL mice. In addition, the transfer of ^45^Ca into the blood was markedly reduced. The calcium concentration in the feces was significantly increased and the gene expression levels of claudin 2 and claudin 15, which are involved in calcium transport, were reduced. No change in the plasma 1,25(OH)_2_D concentration, however, was observed. In the OCL mice, we observed the suppression of a vitamin D-responsive gene (claudin 2) involved in calcium absorption, but no decrease in TRPV6 or calbindin D9K mRNA (64). Based on these findings, we suspect that a system other than vitamin D metabolism is involved in the abnormal intestinal mineral absorption in OCL mice. Although the reasons for the abnormal intestinal mineral absorption remain unknown, osteocytes are considered to be extremely important for intestinal Pi and calcium absorption.

To elucidate the mechanism for abnormal mineral absorption in the OCL mice, we performed DNA microarray analyses of the intestine and liver. We observed alterations in the genes involved in fat and bile acid metabolism (Figures S1 and S2 in Supplementary Material). Metabolic analysis of the OCL mice and G-CSF-treated mice suggested a functional abnormality of the FGF15–FXR–CYP7A1 system in bile acid metabolism (43). Indeed, we observed increased serum bile acid and feces bile acid levels in the OCL mice. A recent study demonstrated that feeding of a lithocholic acid-rich diet increased intestinal Pi absorption and suppressed renal Pi absorption in WT mice (American Society of Nephrology, Kidney Week 2017 Abstract FR-PO258, Hashimoto N, Sakaguchi Y, Hamano T, Isaka Y, Matsui I, Mori D, Matsumoto A, Shimada K, Yamaguchi S, Kubota K, Oka T, Yonemoto S). Lithocholic acid is a known VDR ligand (65). The details of these mechanisms are unknown. However, bile acid metabolism is considered to be a regulator of important for intestinal and renal Pi absorption (66). Bile acids are natural detergents that may solubilize calcium-phosphate particles in the small intestine (67). Bile acids are also important signaling molecules and have a role in energy metabolism, metabolic syndrome, obesity, and diabetes (68). Because Pi is deeply involved in energy metabolism, it is expected to be involved in the regulation of energy metabolism by bile acids.

We investigated whether the enhanced intestinal Pi absorption is linked to changes in FGF15/CYP7A1/bile acid in the OCL mice. Dietary Pi load is a powerful factor to suppress intestinal Npt2b protein levels (42). We investigated the possibility that the FGF15/CYP7A1 system responds to the dietary Pi load. Based on quantitative PCR analysis, the increased gene expression levels in the FGF15–FXR–CYP7A1 system observed in OCL mice were largely suppressed in control mice fed a HP diet. These findings suggest that dietary Pi levels affect the FGF15–FXR–CYP7A1 system in the intestines and liver, in addition to vitamin D metabolism.

Previous studies showed that vitamin D exerts a negative feedback on bile acid synthesis by decreasing Cyp7a1 expression (69, 70). FGF15 and SHP play central roles in the feedback regulation of bile acid synthesis by bile acids and FXR (46–48). FGF15 is integral to the mechanism of CYP7A1 regulation of vitamin D (69, 71). VDR regulates FGF15 in the intestine and this pathway is essential for the vitamin D-induced suppression of bile acid synthesis (72). In the liver, the FGF15/FGFR4/β-klotho complex suppresses SHP expression and increases Cyp7A1 (47, 49, 50). The molecular mechanisms of the vitamin D and bile acid abnormalities in OCL mice are not clear. We speculate that OCL mice have disturbed vitamin D and bile acid (FGF15/FXR/CYP7A1) metabolism due to disruptions of the FGF23–Klotho system (Figure 7).

In conclusion, the findings of the present study suggest that osteocyte ablation stimulates intestinal Pi absorption and enhances renal Pi excretion. The phosphaturic factors active in OCL mice are unknown, but increased Pi absorption is prominent in OCL mice. The marked increase in intestinal Pi absorption may be due to disruption of the FGF23–Klotho system by osteocyte ablation affecting bile acids and vitamin D metabolism. Further studies are needed to clarify the factors involved in the increased renal Pi excretion in OCL mice.

## Author Contributions

ST and K-iM contributed to the study design. OF, MO, TA, HS, KN, AM, KI, AH, IK, HS, ST, and K-iM collected and analyzed data. ST and K-iM wrote and revised the final draft of the manuscript.

## Conflict of Interest Statement

The authors declare that the research was conducted in the absence of any commercial or financial relationships that could be construed as a potential conflict of interest.
