# Supplementary material for: Effect of Osteocyte-Ablation on Inorganic Phosphate Metabolism: Analysis of Bone–Kidney–Gut Axis
Source: Front Endocrinol (Lausanne). 2017 Dec 21;8:359. doi: 10.3389/fendo.2017.00359 (PMC5742590; doi:10.3389/fendo.2017.00359)
Supplement: Supplementary file 1 [file Data_Sheet_1.pdf]

Supplementary Material

Effect of osteocyte-ablation on inorganic phosphate metabolism: analysis of bone-kidney-gut axis

Osamu Fujii, Sawako Tatsumi*, Mao Ogata, Tomohiro Arakaki, Haruna Sakaguchi, Kengo Nomura, Atsumi Miyagawa, Kayo Ikuta, Ai Hanazaki, Ichiro Kaneko, Hiroko Segawa and Ken-ichi Miyamoto*

Correspondence: Sawako Tatsumi: tatsumi@tokushima-u.ac.jp
Ken-ichi Miyamoto: kmiyamoto@tokushima-u.ac.jp

SUPPLEMENTARY METHODS

Determination of bile acid content in mouse small intestine epithelial cells and feces

Tissue (30 mg) was homogenized with 1.0 ml of 70% ethanol and incubated at 55°C for 4 h. The ethanol extracts were evaporated to dryness and resuspended in 300 µl of 0.5-M phosphate buffer (pH 7.0) (1). To determine fecal bile acid excretion, feces from individually housed mice were collected over a 72-h period, weighed, and dried. Then, 40 mg of dried feces was minced and extracted in 800 µl of 75% ethanol at ~50°C for 2 h. The extract was centrifuged, and 100-µl samples of supernatant were diluted to 400 µl with a 25% PBS solution for assay (2). Concentrations of total bile acids were determined using the Total Bile Acid Test Wako (Wako).

Analysis of serum triglyceride and cholesterol levels

Serum triglyceride and cholesterol levels were determined by LabAssay™ Triglyceride (Wako), LabAssay™ Cholesterol (Wako).

SUPPLEMENTARY FIGURES

Supplementary Figure 1. Gene expression profiles and concentration of bile acid cholesterol and triglyceride in OCL mice

Ten-week-old DMP-1-hHB-EGF Tg mice and wild-type mice as littermate controls were injected with DT (50 µg/kg body weight). DT-injected Tg mice are indicated as OCL mice. DT-injected wild-type mice are indicated as control (Cont) mice. A) Photograph of representative kidney in Cont and OCL mice. White adipose tissue around the kidney (mg)/body weight (g) (n=6/group)

(B) and (C) At 5 days after DT injection, total RNA of the liver and distal intestine (late jejunum and ileum) were isolated from OCL and Cont mice. Expression levels of bile acid-related genes were examined using real-time PCR. Data were normalized to GAPDH and pooled from three independent experiments.

B) Liver: Cyp7a1, cholesterol 7α-hydroxylase; FXR, nuclear receptor farnesoid X receptor; Cyp8b1, sterol 12α-hydroxylase; SHP, small heterodimer partner; FGFR4, fibroblast growth factor receptor 4; β-klotho; NTCP, Na^+-taurocholate transporting polypeptide.

C) Distal intestine: IBABP, ileal bile acid binding protein; ASBT, apical sodium-dependent bile acid transporter; FXR; SHP; OST-α, organic solute transporter alpha; OST-β, organic solute transporter beta; FGF15, fibroblast growth factor 15; UGT1a1, UDP glucuronosyltransferase family 1 member A1; UGT1a6, UDP glucuronosyltransferase family 1 member A6; UGT1a7c, UDP glucuronosyltransferase 1 family, polypeptide A7C; UGT2b34, UDP glucuronosyltransferase 2 family, polypeptide B34. The bar graphs are presented as arithmetic means ± SEM (n=6/group). Two-tail unpaired t test $**P<0.01$, $*P<0.05$ vs Cont

D) At 5 days after DT injection, the epithelial cells of mouse small intestine and serum were collected. The feces were collected for 24 h using a metabolic cage. Serum, small intestine and feces bile acid levels were measured. Serum cholesterol and serum triglyceride levels were measured. The bar graphs are presented as arithmetic means ± SEM (n=6/group). Two-tail unpaired t test $**P<0.01$, $*P<0.05$ vs Cont

Supplementary Figure 2.

Gene expression profiles and bile acid cholesterol and triglyceride concentrations in high Pi diet-fed mice.

Ten-week-old wild-type mice as littermates were fed the Control Pi diet (CP) or the High Pi diet (HP) for 1 week. A) Photograph of a representative kidney from CP-fed and HP-fed mice. White adipose tissue around kidney (mg) / body weight (g) (n=6/group)
(B) and (C) Total RNA of the liver and distal intestine (late jejunum and ileum) were isolated from CP and HP mice. Expression levels of bile acid-related genes were examined using real-time PCR. Data were normalized to GAPDH and pooled from three independent experiments. (n=6/group)
B) Liver: Cyp7A1, cholesterol 7α-hydroxylase; FXR, nuclear receptor farnesoid X receptor; Cyp8b1, sterol 12α-hydroxylase; SHP, small heterodimer partner; FGFR4, fibroblast growth factor receptor 4; β-klotho; NTCP, Na^+-taurocholate transporting polypeptide.
C) Distal intestine: IBABP, ileal bile acid binding protein; ASBT, apical sodium-dependent bile acid transporter; FXR; SHP; OST-α, organic solute transporter alpha; OST-β, organic solute transporter beta; FGF15, fibroblast growth factor 15; UGT1a1, UDP glucuronosyltransferase family 1 member A1; UGT1a6, UDP glucuronosyltransferase family 1 member A6; UGT1a7c, UDP glucuronosyltransferase 1 family, polypeptide A7C; UGT2b34, UDP glucuronosyltransferase 2 family, polypeptide B34.
D) Epithelial cells of mouse small intestine and serum were collected. Serum and small intestine bile acid levels were measured. Serum cholesterol and serum triglyceride levels were measured. The bar graphs are presented as arithmetic means ± SEM (n=6/group). Two-tail unpaired t test $**P<0.01$, $*P<0.05$. (CP VS HP)

Supplemental Table 1. Primer sequences for real-time RT-PCR

Gene -Bone	Forward primer	Reverse primer	Product size (bp)
DMP1	5'-GGCTGTCCTGTGCTCTCCCAG-3'	5'-GGTCACTATTTGCCTGTGCCTC-3'	159
E11/GP38	5'-CAGTGTTGTTCTGGGTTTTGG-3'	5'-GGGGTCACAATATCATCTTCA-3'	91
MEPE	5'-GTGAATGACGCCAGAGGGC -3'	5'-TGTCTTCATTCGGCATTGG-3'	98
Cbfa1	5'-CACTGCCACCTCTGACTTCT -3'	5'-GCTCTCAGTGAGGGATGAAA -3'	127
ALP	5'-CAGGGTACACCATGATCTCACC-3'	5'-CGCCCATACCATCTCCCAGG -3'	180
Osteocalcin	5'-GAGGACCATCTTTCTGCTCAC-3'	5'-CCAAGGTAGCGCCGGAGTCTG- 3'	153
FGF23	5'- ACTTGTCGCAGAAGCATC-3'	5'-GTGGGCGAACAGTGTAGAA-3'-3'	144
SOST	5'-GGAATGATGCCACAGAGGTCA -3'	5'-CCCGGTTCATGGTCTGGTTT -3'	81
Phex	5'--GTGCATCTACCAACCAGATACG-3'	5'-TCTGTTCCCCAAAAGAAAGG-3'	67
Osteopontin	5'-GTGAAAGTGACTGATTCTGGCAGC-3'	5'-CATCATCGTCGTCCATGTGGTCAT-3'	214
Col 1a1	5'-TCCCTGAAGTCAGCTGCATA-3'	5'-TGGGACAGTCCAGTTCTTCAT-3'	144
Gene -Liver	Forward	Reverse	
FGFR4	5'-CGCATGGAGAAGAAACTGCA-3'	5'-CCTGTCCATCCTTGAGCCAG-3'	109
FXR	5'- GCACGCTGATCAGACAGCTA -3'	5'-CAGGAGGGTCTGTTGGTCTG-3'	121
NTCP	5'-TGAAGGGGGACATGAACC-3'	5'-GTAGATGTATAAGAGGAGAGGCATCA-3'	89
Cyp7a1	5'-AGCAACTAAACAACCTGCCAGTACTA-3'	5'-GTCCGGATATTCAAGGATGCA -3'	82
Cyp8b1	5'-ACGCTTCCTCTATCGCCTGAA-3'	5'-GATGGGTATTCAGTGCTGC-3'	84
SHP	5'-CGATCCTCTTCAACCCAGATG-3'	5'-AGGGCTCCAAGACTTCACACA-3'	102
β-Klotho	5'-TGGGGAGTCACTGAGTCTGT-3'	5'-CATACAGGTGAGGATCGGTAAAC-3'	76
Gene–intestine	Forward	Reverse	
TRPV6	5'-CTCCTCATTGCCATGATGGG -3'	5'-GGCCACAACCTGTGCTCTCCAG-3'	81
CaBP D9k	5'-CCTGCAGAAATGAAGAGCATTTT-3'	5'-CTCCATCGCCATTCTTATCCA-3'	172
PMCA1b	5'-CGCCATCTTCTGCACCATT-3'	5'-CAGCCATTGCTCTATTGAAAGTTC -3'	109
Claudin2	5'-ATACTACCCTTTAGCCCTGACCGAGA-3'	5'-CAGTAGGAGCACACATAACAGCTACCAC-3'	95
Claudin12	5'-CAGACCAGTGTGTACTCAGACTTTCTACCC-3'	5'-GAAGCAACATACTGACTGTCTCCTGACG-3'	109
Claudin15	5'-CATCTTTGAGAACCTGTGGTACAGC-3'	5'-GATGGCGGTGATCATGAGAGC-3'	130
OST-α	5'- ATGCATCTGGGTGAACAGAA -3'	5'-GAGTAGGGAGGTGAGCAAGC-3'	134
OST-β	5'-GACCACAGTGCAGAGAAAGC-3'	5'-CTTGTCATGACCACCAGGAC-3'	142
ASBT	5'-GGAACTGGCTCCAATATCCTG-3'	5'-GTTCCCGAGTCAACCCACAT-3'	146
FGF15	5'-GAGGACCAAAACGAACGAAA-3'	5'-ACGTCCTTGATGGCAATCG-3'	71
IBABP	5'-GGTCTTCCAGGAGACGTGAT-3'	5'-ACATTCTTTGCCAATGGTGA-3'	144
UGT1a1	5'-ATGGCTTTCTTCTCCGGAAT-3'	5'-CAGAAAAAGCCCCTATCCC-3'	116
UGT1a6	5'-CACCGGAACTAGACCATCGAA-3'	5'-GCATCATCACCATCGGAACTC-3'	164
UGT1a7c	5'-TGCAATGGAGTTCCGATGGT-3'	5'-CTGGAGAGGCGCATGATGTT-3'	188
UGT2b34	5'-GGAGAATGCCATGCGGTTAT-3'	5'-CTGCCACACGAAGATGCTTG-3'	122
Gene–kidney	Forward	Reverse	
Npt2a	5'-AGAGCCCTTCACAAGACTCATCAT-3'	5'-TACCCTGGACATAGAAGTGGAAGC-3'	148
Npt2c	5'-TGAAGAACGCTGACCAACTGA-3'	5'-AGCAGAGCTGAGGATGTCCAG-3'	137
Pit2	5'-CTCAGAAGGCACGTCAGCAG -3'	5'-AAACGTGACCGTCATTCCTC-3'	140
1α(OH)ase	5'-GAGCAAACTCCAGGAAGCAG-3'	5'-TGAGGAATGATCAGGAGAGG-3'	114
24(OH)ase	5'-TGGGAAGATGATGGTGACCC-3'	5'-TCGATGCAGGGCTTGACTG -3'	114
α-klotho	5'-AATTATGTGAATGAGGCTCTGAAAG-3'	5'-TACGCAAAGTAGCCACAAAGG-3'	68
CaSR	5'-TGCAGACATCAAGAAGGTTGA-3'	5'-CCGCACTCATCGAAGGTC-3'	102
TRPV5	5'-GTTGGTTCTTACGGGTTGAAC-3'	5'-CTGCTCTTGTACTTCCTC-3'	104
Egr-1	5'-AGCCGAGCGAACAACCCTAT-3'	5'-CGCCTTCTCATTATTCAGAGCG-3'	74
FGFR1	5'-CTGGAGTTAATACCACCGAC-3'	5'-TGCAGAGTGATGGGAGAGTC-3'	122
FGFR3	5'-GCACAACCTGGACTACTACAAG-3'	5'-CAGCGTAAAGATCTCCCAGAG-3'	145
Internal Control	Forward	Reverse	
GAPDH	5'-CTGCACCACCAACTGCTTAGC-3'	5'-CATCCACAGTCTTCTGGGTG-3'	116

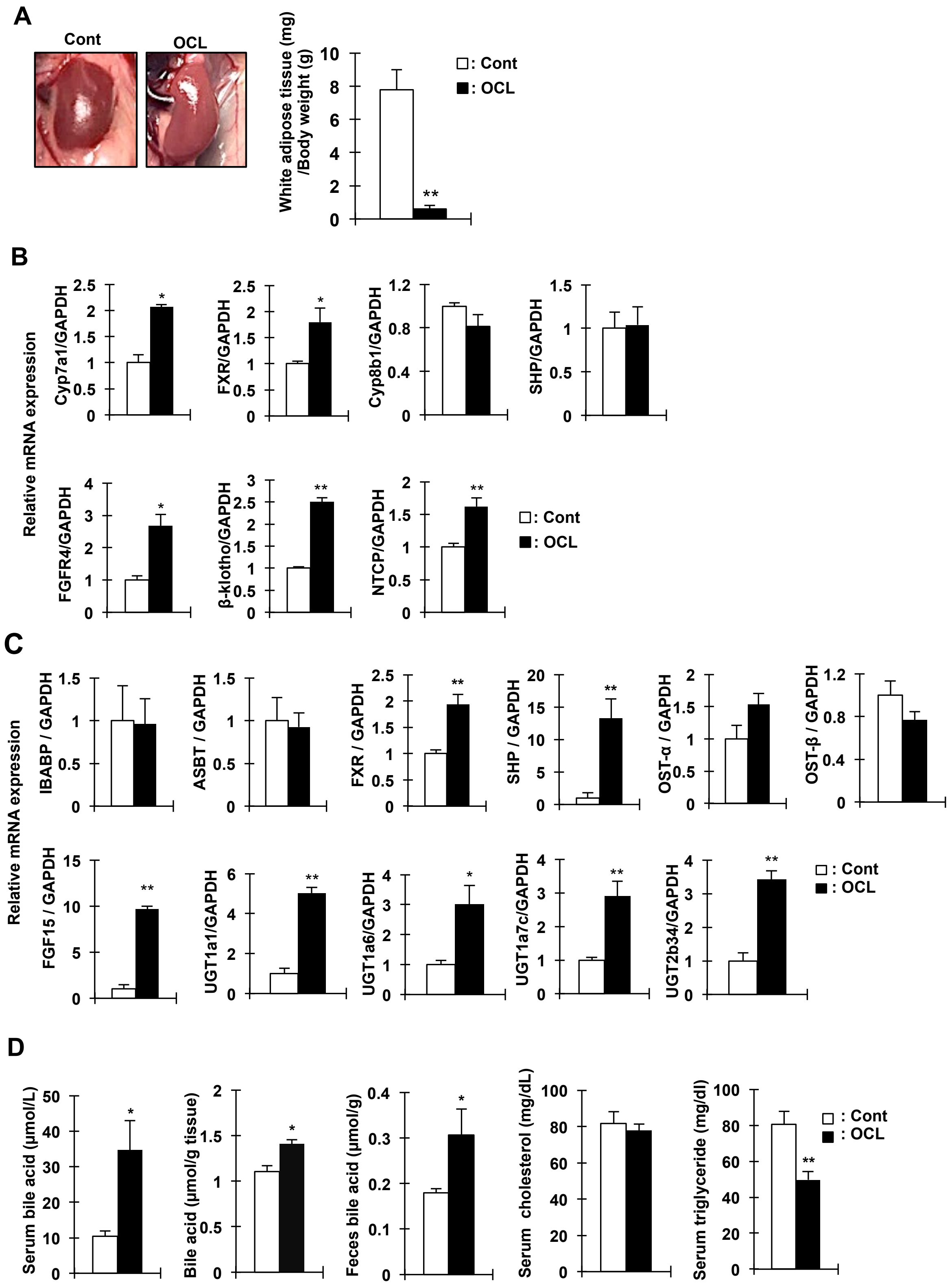

Supplementary Figure 1

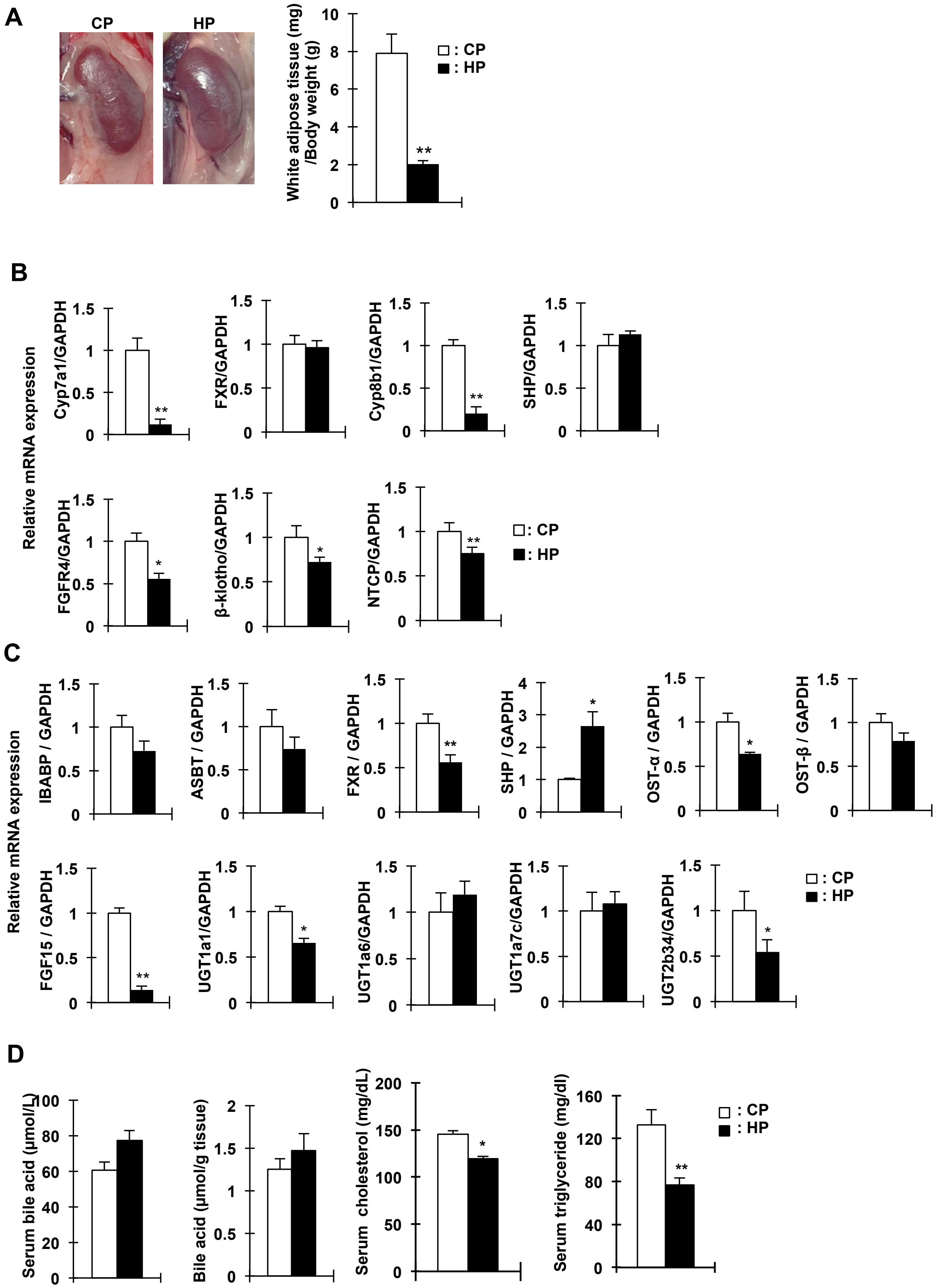

Supplementary Figure 2
